# Saliva MicroAge: A salivary microbiome based machine learning model for noninvasive aging assessment and health state prediction

**DOI:** 10.1002/imo2.70040

**Published:** 2025-07-08

**Authors:** Tiansong Xu, Yuting Niu, Chenyu Deng, Yoo Cheung, Yuman Li, Zhewen Hu, Shiyu Sun, Yiming Chen, Fan He, Gai Yang, Feng Chen, Chenggang Duan, Ying Huang, Xuliang Deng

**Affiliations:** ^1^ Department of Geriatric Dentistry Peking University School and Hospital of Stomatology & National Center for Stomatology & National Clinical Research Center for Oral Diseases & National Engineering Research Center of Oral Biomaterials and Digital Medical Devices Beijing China; ^2^ Fifth Clinical Division, Peking University School and Hospital of Stomatology & National Center for Stomatology & National Clinical Research Center for Oral Diseases & National Engineering Research Center of Oral Biomaterials and Digital Medical Devices Beijing China; ^3^ Department of Orthodontics Peking University School and Hospital of Stomatology & National Center for Stomatology & National Clinical Research Center for Oral Diseases & National Engineering Research Center of Oral Biomaterials and Digital Medical Devices Beijing China; ^4^ Lingchuan Stomatology Inc. Beijing China; ^5^ Central Laboratory, Peking University School and Hospital of Stomatology & National Center for Stomatology & National Clinical Research Center for Oral Diseases & National Engineering Research Center of Oral Biomaterials and Digital Medical Devices Beijing China

## Abstract

Saliva MicroAge is a machine learning‐based model designed to estimate biological age and assess health status using globally sourced salivary microbiome data. Trained on 4532 healthy samples, the model achieves high accuracy in predicting chronological age and captures health‐related deviations (MicroAgeGap) in various diseases. Taxonomic and functional analyses of key microbial features reveal biological relevance to aging processes, offering a noninvasive and scalable approach for aging monitoring and precision health assessment.

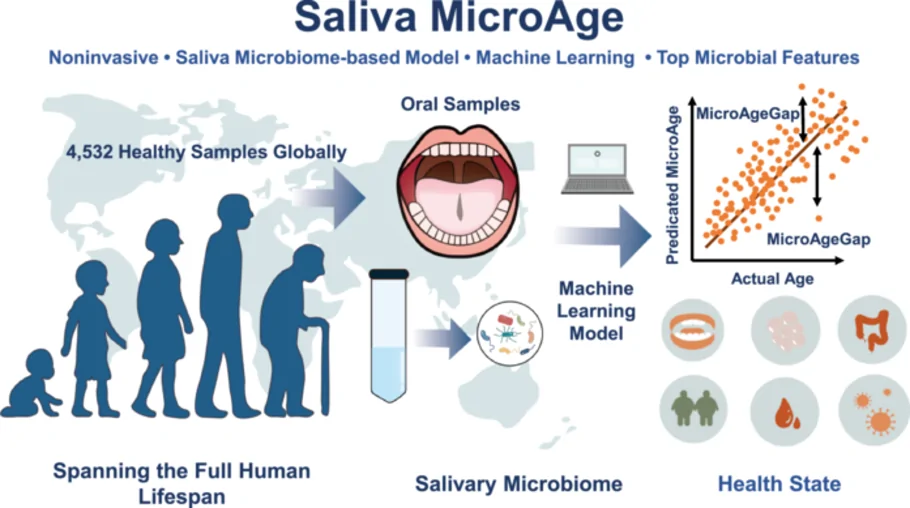

## ETHICS STATEMENT

No animals or humans were involved in this study.

To the Editor,

Aging is a major determinant of chronic diseases and mortality, leading to a gradual decline in physiological function and increased comorbidities such as cancer, cardiovascular disease, and neurodegeneration [1]. With the global population aged 65 and older rising from 6.8% in 2000 to 10.0% in 2023, understanding and predicting the aging process and related diseases have become crucial for addressing the growing healthcare demands [2, 3, 4].

With the emergence of various predictive methods, such as machine learning, researchers across multiple fields have made significant efforts in developing multi‐level aging prediction models. However, current predictive models still face numerous challenges, including invasiveness, high costs, and limited data inclusion. Some predictive methods were invasive, expensive, or limited in data inclusion. To overcome these issues, it is essential to explore noninvasive, convenient, and universally applicable methods for predicting age.

The microbiota is widely distributed throughout the human body and interacts closely with the host immune system, playing a critical role in both the aging process and systemic health [5, 6]. Among these microbial communities, the oral microbiome is the second largest and exhibits remarkable diversity, with its composition and function showing strong associations with age. However, the complex ecology and high diversity of the oral microbiome have made it challenging to clearly elucidate its relationship with aging or to develop accurate age prediction models based on individual microbial features. To address these challenges, we propose a combined approach leveraging microbiome analysis and machine learning. Based on this, we developed a salivary microbial age prediction model (MicroAge) using a large‐scale data set from healthy populations. We further evaluated the relationship of health states on the discrepancy between saliva MicroAge and chronological age (MicroAgeGap). Additionally, we analyzed the functional predictions and microbial networks of the top 30 microbial features to uncover their potential biological significance in aging. Our study offers the potential to predict MicroAge based on saliva microbiota, assess the correlation between MicroAge and health states, and improve clinical prognoses, presenting significant opportunities for advancing precision geriatric medicine.

## 1 RESULTS AND DISCUSSION

### Characteristics of the human saliva samples

**Figure 1:**
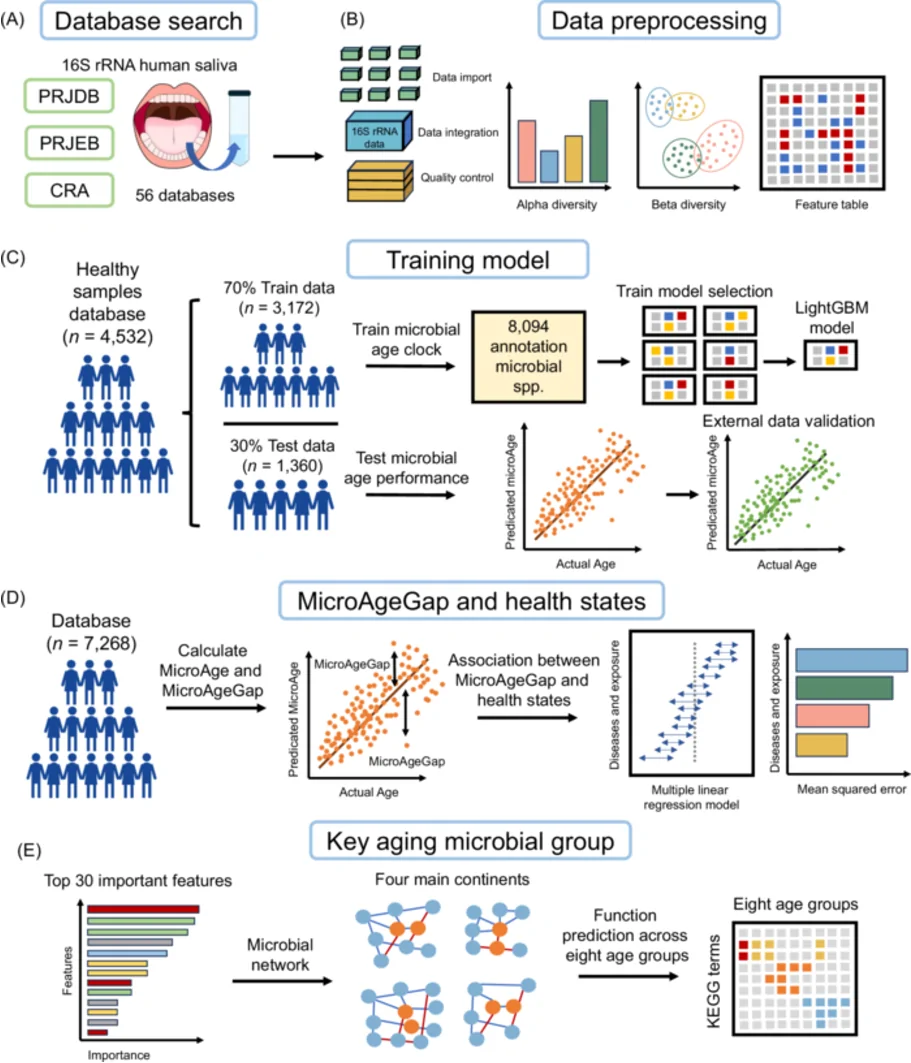
Overview of the study design and analytic approaches. (A) A PubMed search for published studies was performed using the keywords “16S rRNA human saliva” and “16S rRNA saliva.” In total, 56 databases were enrolled in our future analysis. (B) QIIME2 pipeline was applied for data import and preprocessing. The alpha and beta diversity of oral microbiota were computed across the different age groups. A feature table annotated with the Silva reference database was utilized for subsequent analyses. (C) The health samples database (*n* = 4532) was split into training and test sets at a 70:30 ratio. LightGBM model was selected for Microbial predicted age (MicroAge) based on the correlation coefficient between predicted MicroAge and actual age. And the correlation was tested across four continents. (D) MicroAge and MicroAgeGap were calculated in all the samples. MicroAgeGap was calculated as the difference between MicroAge and actual age. We used multiple linear regression to test associations between MicroAgeGap and diseases and exposure. Mean squared error was applied to assess the volatility of MicroAgeGap under different conditions. (E) Top 30 important features were selected based on their Gain values in LightGBM model. The network and function prediction of the top 30 important features were computed to show potential functions.

A schematic overview of the study design and the primary analytical approaches was provided in Figure 1A–E. Sequencing data were obtained from the National Center for Biotechnology Information (NCBI) Sequence Read Archive and databases from our team published previously (a total of 56 databases) focused on massively parallel sequencing of human 16S saliva samples (Table S1).

In total, 7268 samples were collected across six continents, including 493 infants, 1750 children, and 4385 adolescents/adults, ranging from 0 to 107 years (Figure S1A, Table S2). 62.36% samples reported their health to be good or very good. Other samples were involved in several types of diseases and exposure statuses, such as viral infection, Obese related diseases, oral diseases or status (Figure S1B).

### The saliva microbiota across the human lifespan

We utilized the QIIME2 pipeline for data import and preprocessing. All raw sequences were subjected to quality control using a consistent index and subsequently integrated into a unified data set.

To investigate the potential relationship between saliva microbiota and age, we calculated the alpha diversity of oral microbiota across different age groups. Shannon diversity indices increased across the age categories of 0, 1–4, and 5–9 years, followed by a stepwise decline in diversity in 15–24 years old, and another decrease in diversity was seen in over 65 years, indicating that saliva microbiota continues to develop until adolescent, experienced a small fluctuation in young adulthood, and declines in old age (Figure S1C,D). The relative abundances of the main genus changed with age. The percentage of *Streptococcus* was decreasing, while *Prevotella* and *Veillonella* were increasing with age (Table S3, Figure S1E,F). The above results revealed a potential association between saliva microbiota and age‐related changes.

### The construction of saliva MicroAge prediction model

We randomly split the healthy sample databases into 70% training and 30% test sets to develop a saliva MicroAge predicted model. During the training phase (*n* = 4532), we evaluated six machine‐learning methods to train saliva MicroAge predicted models for predicting chronological age using normalized expression composed of 8094 microbiome species (Figure S2). Among these methods, we found that gradient boosting (LightGBM) demonstrated the highest age prediction accuracy in the test set (*R* ^2^ = 0.7983, RMSE = 9.34, MAE = 5.92, Figure S2A, Table S4). The model also showed robust predictive performance across the four main continents in the test set (Figure S2B). Due to its superior generalizability, we selected LightGBM as our final model.

We also evaluated our model using an external data set from PRJNA1162741 [7]. Despite the significant differences in age distribution and no specific identifiers for healthy controls, our model yielded an intriguing result: we identified 136 individuals whose predicted MicroAge fell within ±5 years of their chronological age, a number close to the reported count of healthy controls in the original study (Figure S2C).

### Saliva MicroAge prediction model in diseases and extreme environment states

Next, we applied the saliva MicroAge prediction model to the whole databases, to investigate whether variations exist between salivary MicroAge and chronological age in varying disease conditions and extreme environments. The results revealed a lower correlation between MicroAge and chronological age in unhealthy samples (*R* ^2^ = 0.1642, RMSE = 21.16, MAE = 16.16; Figure S2D), compared to healthy populations.

This trend was consistently observed across specific disease groups, including Head and neck cancer, Schizophrenia, Asthma, and COVID‐19 infection samples (Figure S2E), suggesting that the model captures variance primarily driven by disease‐related factors. Other machine‐learning models are shown in Figure S3.

### The volatility of saliva MicroAge might be a powerful predictor of health states

**Figure 2:**
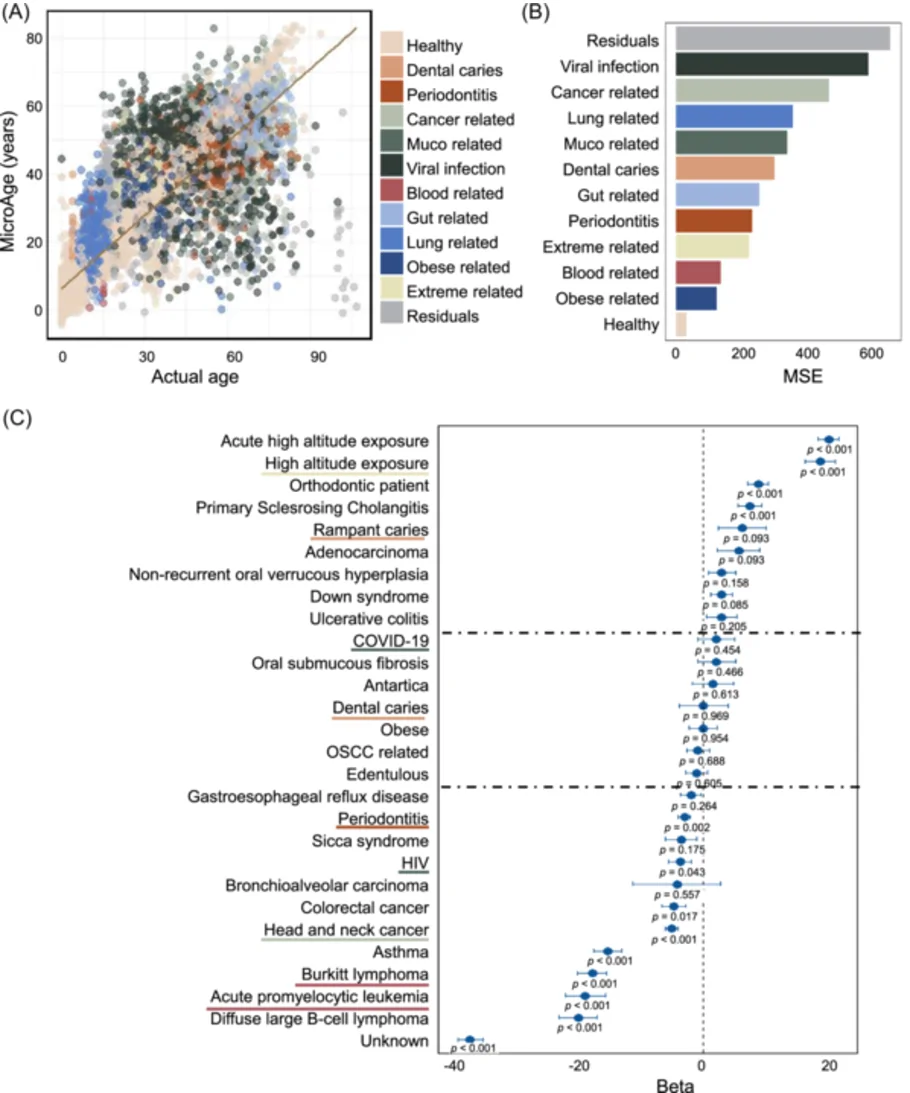
The volatility of MicroAge was associated with diseases and environmental statuses. (A) Scatter plot illustrating the association between predicted MicroAge and actual age, colored by diseases and environmental statuses. (B) Bar plot depicting mean squared error (MSE) of predicted MicroAge compared to actual age across various disease types and environmental conditions. (C) Associations between MicroAgeGap and types of diseases and environmental status from all the samples (*n* = 7235). The models used multiple linear regression and were adjusted for regions, sex, and age groups. The beta estimates (and 95% confidence intervals) for the association between MicroAgeGap and each outcome were shown on the *x*‐axis. Significant results were highlighted with corresponding *p* values. COVID‐19, Corona Virus Disease 2019; HIV, Human Immunodeficiency Virus; MSE, Mean Squared Error; OSCC, Oral Squamous Cell Carcinoma

To understand how saliva microbial aging may influence human diseases and various environmental exposure states, we categorized disease types and exposure environments in the database samples and investigated the association between predicted saliva MicroAge and these states (Figure 2A). Mean squared error (MSE) was applied to evaluate the generalization ability of MicroAge model compared to chronological age under different states. We found that MicroAge exhibited the least fluctuation under healthy states, whereas its variability increased under disease states, suggesting that the model performs most consistently under healthy states. Among all states, the fluctuation of MicroAge was the largest under viral infection, excluding residuals state (Figure 2B). Detailed effect size (Cohen's *d*) results are provided in Table S5. The volatility of saliva MicroAge compared to chronological age may be a powerful predictor of health states.

To further investigate, we defined saliva MicroAgeGap as the difference between saliva MicroAge and chronological age, and further used multiple linear regression models to investigate whether associations of MicroAgeGap with different health states included in our database (Figure 2C). Healthy state was set as the reference benchmark. The forest plots were divided into three sections by two dashed lines according to regression coefficient. The states in the upper section have positive regression coefficients, indicating a positive effect on predicted age. These states were associated with inflammation, such as rampant caries, ulcerative colitis, and adenocarcinoma. The middle section represents conditions with minimal impact on predicted age, including diseases such as dental caries and oral submucosal fibrosis. The lower section contains diseases with negative regression coefficients, indicating a negative effect on predicted age. Many of these were related to immune deficiencies, such as HIV and diffuse large B‐cell lymphoma.

Our findings suggested that a positive increase in saliva MicroAgeGap relative to the healthy baseline may be linked to aging in inflammatory states, whereas a negative increase may indicate a decline in immune function. MicroAgeGap might be a powerful predictor of health states.

### The association between MicroAgeGap and the severity of oral diseases

Then, we explored the association between MicroAgeGap and oral diseases and focused on two important oral diseases: dental caries and periodontitis [8, 9, 10].

For dental caries, the fluctuation of MicroAgeGap in common dental caries and rampant caries was the smallest, while the fluctuation of arrested caries and rampant caries accompanied by high‐sugar diet was the largest (Figure S4A).

For periodontitis, severe periodontitis with HIV infection had the highest fluctuation of MicroAgeGap, and there was a statistically significant difference in MicroAgeGap between the severe periodontitis and healthy groups (Figure S4B). In addition, we found that as the severity of periodontitis increased, MicroAgeGap also gradually increased (Figure S4C).

### Lower MicroAge predicted in centenarians

Subsequently, we investigated the connection between MicroAge and exceptional longevity. We collected and downloaded 64 saliva samples from the PRJEB25916 data set (Figure S5A), which included a younger, elder age, and centenarian groups (Figure S5B–D) [11]. Centenarian group had a larger MicroAgeGap compared to both the younger and elder age groups, meaning that their predicted age was considerably younger than their actual age. The results suggest that centenarians have a protective MicroAge phenotype, indicating that MicroAge is associated with exceptional longevity and might provide a more accurate indication of true biological age compared to chronological age [12].

### Top 30 important features of saliva MicroAge predicted model

To determine which microbial species were most critical for predicting chronological age among all microbial species included, we identified the top 30 most important features in the LightGBM model. The most important feature was *Parvimonas micra*, an oral opportunistic pathogen frequently associated with oral diseases such as periodontitis, apical periodontitis [13, 14, 15]. Notably, some species with relatively low abundances in saliva, such as *Porphyromonas gingivalis*, were also among the top 30 important features and closely linked to diseases like periodontitis [16, 17], highlighting the clinical relevance of our model (Figure S6A).

Then, we used the top 30 features to construct a prediction model, and the model demonstrated robust predictive performance (Figure S6B). Furthermore, when applying the top 30 features in a multiple linear regression model to predict MicroAgeGap in relation to diseases and environmental exposures, the simplified model achieved a correlation coefficient similar to the full‐feature model, further validating its utility (Figure S6C). In the microbial networks, we observed that top 30 important features consisently linked with other features across four continents (Figure S6D).

We predicted the potential functions of the top 30 important features using Tax4Fun (Figure S7). In the 0–9 age group, the functions of the top 30 important features were associated with terms such as metabolism, genetic information processing. In the middle‐aged and elderly groups, the functions of the top 30 important features were linked to aging, oxidative phosphorylation, and immune diseases. Our findings suggest that the core salivary microbiota might have a role in co‐developing alongside human biological processes.

Our study analyzed 7268 16S salivary microbiome samples from six continents, assessing microbial diversity and abundance across different age groups to explore their relationship with age. We then developed and validated a machine learning model to predict saliva MicroAge, demonstrating strong predictive accuracy in healthy populations. We also investigated the variability between MicroAge and chronological age (MicroAgeGap), revealing its association with various health states.

Our approach offers several advantages, including the use of machine learning models and microbiome data, which effectively capture nonlinear relationships and interactions within the oral microbiome. Additionally, our model evaluation demonstrates that our microbial age prediction model significantly outperforms traditional methods, providing enhanced accuracy and generalizability in predicting biological age compared to other algorithms like LASSO and elastic net [2].

However, there were still some limitations in our study, and future research directions to investigate. First, the sample size remained relatively small. In future research, we aimed to expand our database to develop a more robust model. Second, our data were from the NCBI database. Several specific sample information, such as saliva sample storage, time, environmental factors and preprocessing, antibiotic use, and social relationships, remained unknown. In future studies, we will integrate clinical research and experimental approaches to explore the mechanistic explanation in greater depth. Large language models will be applied in our prediction model due to their impressive capabilities.

## 2 CONCLUSION

In conclusion, our study demonstrates that salivary microbiomes are valuable for predicting age. The discrepancies between chronological age and saliva MicroAge were linked to health states. Moreover, the key microbial species identified in our model highlight their biological significance and potential as therapeutic targets for diagnosing and managing aging‐related conditions. These findings offer promising applications in predicting aging and health states, as well as advancing personalized healthcare and microbiome research.

## 3 METHODS

Detailed procedures for oral microbiome data analysis and construction of mechanical learning models are available in the Supplementary Information.

## AUTHOR CONTRIBUTIONS

**Tiansong Xu**: Conceptualization; data curation; formal analysis; investigation; methodology; software; visualization; writing—original draft. **Yuting Niu**: conceptualization; methodology; software; data curation; investigation; formal analysis; visualization; writing—original draft. **Chenyu Deng**: Conceptualization; methodology; software; data curation; investigation; formal analysis; visualization; writing—original draft. **Yoo Cheung**: Validation; formal analysis; resources. **Yuman Li**: Validation; formal analysis; resources. **Zhewen Hu**: Validation; formal analysis; resources. **Shiyu Sun**: Validation; formal analysis; resources. **Yiming Chen**: Validation; formal analysis; resources. **Fan He**: Validation; formal analysis; resources. **Gai Yang**: Validation; formal analysis; resources. **Feng Chen**: Methodology. **Chenggang Duan**: Conceptualization; funding acquisition; project administration; writing—review and editing; resources; supervision. **Ying Huang**: Conceptualization; writing—review and editing; resources; project administration; funding acquisition; supervision. **Xuliang Deng**: Conceptualization; writing—review and editing; resources; project administration; funding acquisition; supervision.

## CONFLICT OF INTEREST STATEMENT

The authors declare no conflicts of interest.

## Supporting information


**Figure S1.** Alterations of the saliva microbiota across age groups.
**Figure S2.** Performance of the trained microbial aging model and external validation.
**Figure S3.** Microbial aging performance based on the other selected machine‐learning Model algorithm.
**Figure S4.** The fluctuation of MicroAgeGap was correlated with the severity of oral diseases.
**Figure S5.** MicroAge in younger, older age and centenarian samples.
**Figure S6.** Top 30 important features contributing to MicroAge predicted model.
**Figure S7.** The function prediction of top 30 important features across the lifespan.

**Table S1.** The databases downloaded and collected in our databases.
**Table S2.** The distribution of samples across continents and age groups.
**Table S3.** The microbial phylum distribution of samples from different age groups.
**Table S4.** Performance comparison of six machine learning algorithms in the construction of the MicroAge prediction model.
**Table S5.** Cohen's *d* values comparing predicted age and actual age across different health state groups.

## Data Availability

Raw 16S rRNA gene sequencing data were available with the accession numbers shown in Table S1. Supporting code and raw data were provided at https://github.com/willmaxu/Saliva-MicroAge/, and name and versions of the software were included in the method. Supplementary materials (methods, figures, tables, graphical abstract, slides, videos, Chinese translated version, and update materials) may be found in the online DOI or iMetaOmics http://www.imeta.science/imetaomics/.
