# Supplementary material for: Saliva MicroAge: A salivary microbiome based machine learning model for noninvasive aging assessment and health state prediction
Source: IMetaOmics. 2025 Jul 8;2(4):e70040. doi: 10.1002/imo2.70040 (PMC12806003; doi:10.1002/imo2.70040)
Supplement: Supplementary file 1 — Figure S1. Alterations of the saliva microbiota across age groups. Figure S2. Performance of the trained microbial aging model and external validation. Figure S3. Microbial aging performance based on the other selected machine‐learning Model algorithm. Figure S4. The fluctuation of MicroAgeGap was correlated with the severity of oral diseases. Figure S5. MicroAge in younger, older age and centenarian samples. Figure S6. Top 30 important features contributing to MicroAge predicted model. Figure S7. The function prediction of top 30 important features across the lifespan. [file IMO2-2-e70040-s002.docx]

**Supporting information to**

**Saliva MicroAge: A Salivary Microbiome-based Machine Learning Model for Noninvasive Aging Assessment and Health State Prediction**

Running title: Saliva MicroAge: Predicting Aging and Health from Oral Microbiota

Tiansong Xu^1,2#^, Yuting Niu^1#^, Chenyu Deng^1,3#^, Yoo Cheung^4^, Yuman Li^1^, Zhewen Hu^1^, Shiyu Sun^1^, Yiming Chen^1^, Fan He^1^, Gai Yang^1^, Feng Chen^5^, Chenggang Duan^2*^, Ying Huang^1*^, Xuliang Deng^1*^

1 Department of Geriatric Dentistry, Peking University School and Hospital of Stomatology & National Center for Stomatology & National Clinical Research Center for Oral Diseases & National Engineering Research Center of Oral Biomaterials and Digital Medical Devices, Beijing, 100081, China

2 Fifth Clinical Division, Peking University School and Hospital of Stomatology & National Center for Stomatology & National Clinical Research Center for Oral Diseases & National Engineering Research Center of Oral Biomaterials and Digital Medical Devices, Beijing, 100081, China

3 Department of Orthodontics, Peking University School and Hospital of Stomatology & National Center for Stomatology & National Clinical Research Center for Oral Diseases & National Engineering Research Center of Oral Biomaterials and Digital Medical Devices, Beijing, 100081, China

4 Lingchuan Stomatology Inc., Beijing, 100176, China

5 Central Laboratory, Peking University School and Hospital of Stomatology & National Center for Stomatology & National Clinical Research Center for Oral Diseases & National Engineering Research Center of Oral Biomaterials and Digital Medical Devices, Beijing, 100081, China

# These authors contributed equally: Tiansong Xu, Yuting Niu, Chenyu Deng

*Corresponding Authors: kqdengxuliang@bjmu.edu.cn (Xuliang Deng); hying031129@163.com (Ying Huang); chenggang93@sina.com (Chenggang Duan)

**Supplementary methods**

**Data collection**

We conducted a PubMed search for published studies using the keywords “16S rRNA human saliva” and “16S rRNA saliva” up to October 5, 2024. Only studies that provided raw 16S rRNA gene sequencing data and accompanying patient information, such as age, disease state, were considered for inclusion in our analysis. After a comprehensive search and eligibility evaluation, 56 raw 16S rRNA gene sequencing datasets from human saliva across six continents were selected. The raw sequencing data were downloaded using SRA tools and the accession numbers listed in Dataset S1 [1-11].

**16S rRNA data analysis**

The raw sequencing reads of 16S rRNA were processed and assessed using QIIME2 (v 2021.4) [12], with the Silva reference database (version 132) employed for annotating oral microbial genomes [13]. Sequencing data were processed using the DADA2 plugin within QIIME2 for quality control and denoising. Forward and reverse reads were truncated at 250 base pairs to remove low-quality sequence tails, and the first 12 bases of both forward and reverse reads were trimmed to eliminate primers and improve sequence quality. After merging feature tables across studies, we normalized each sample by converting feature counts into relative abundances (0–1 scale) to account for differences in sequencing depth.

Alpha diversity metrics were derived from the QIIME2 pipeline, while beta diversity was computed using the diversity and PCoA functions from the R packages vegan (v2.6-2) and ape (v5.6-2). Data visualization was performed with the R package ggplot2 (v3.4.4). Finally, a reference feature table generated by QIIME2 was utilized for subsequent analyses.

**Machine-learning Model Algorithms**

Six machine-learning algorithms were employed for model construction: Lasso regression (LASSO) using the R package glmnet (v4.1-8) [14], eXtreme Gradient Boosting (XGBoost) with the R package xgboost (v1.7.7.1) [15], Light Gradient Boosting Machine (LightGBM) with the R package Lightgbm (v4.5.0)[16], Elastic Net using the R package glmnet[17], a simple neural network implemented with the R package nnet (v7.3-14) [18], and a deep neural network using the R package Keras (v2.15.0) [19]. Subsequently, 54 imputed datasets were generated, each corresponding to specific clinical outcomes and patient treatment groups. The six machine-learning algorithms were applied to each dataset, and the results for each dataset were summarized individually.

We implemented six machine learning models for age prediction based on salivary microbiome features. Hyperparameter tuning was conducted for each model as follows:

- XGBoost: The model was trained with the following parameters: eta = 0.3, gamma = 0.001, max_depth = 2, subsample = 0.7, colsample_bytree = 0.4, nrounds = 2000, and early_stopping_rounds = 1000.
- Keras Neural Network: A simple neural network was trained using the following setup:epochs = 100, batch_size = 32, and validation_split = 0.2.
- LASSO: We applied 10-fold cross-validation to identify the optimal lambda parameter using alpha = 1.
- Elastic Net: Cross-validation was performed with alpha = 0.5 to select the best lambda.
- Simple Neural Network: Prior to modeling, PCA was applied to reduce dimensionality to the top 150 principal components. The neural network was trained with size = 5 and maxit = 200.
- Decision Tree Algorithm (LightGBM): The LightGBM model was trained with:num_leaves = 31, learning_rate = 0.05, feature_fraction = 0.9, and early_stopping_rounds = 10.

**Model evaluation indexes**

The correlation coefficient between predicted microAge and actual age was used as the primary index for assessing model performance. Additionally, other performance metrics, including the *p*-value, Root Mean Squared Error (MSE) and Mean Absolute Error (MAE) [20, 21], were thoroughly evaluated. The correlation coefficient was computed using Pearson's test via the functions ‘cor’ and ‘cor.test’.

**Associations between MicroAgeGap and diseases**

All associations between MicroAgeGap and diseases or exposure states were evaluated using a multiple linear regression model. In this model, independent variables such as regions, sex, and age groups were excluded to minimize potential confounding effects. The results were further processed to extract model terms and their corresponding statistics. A forest plot was created to illustrate the estimated effects (Beta) of various diseases on MicroAgeGap. The plot features point estimates accompanied by 95% confidence intervals, displayed as horizontal error bars. Significant results are highlighted with *p*-values.

**Selection of Top 30 important features**

The feature importance of the trained LightGBM model was calculated using the ‘lgb.importance’ function, with the importance scores expressed as percentages. From this, the top 30 features were selected based on their Gain values, which measure each feature contribution to the performance of model. The names of these top features were extracted and stored for further analysis, highlighting the most influential predictors in the model.

**The network and function prediction of Top 30 important features**

Networks among the top 30 important features and other features were constructed based on correlations with absolute Spearman’s coefficients > 0.6 and *p*-values < 0.05, using the R package corrplot (v0.90). The microbial community network diagrams were computed and visualized using the interactive platform Gephi (v0.9.2) [22].

Functional predictions of 16S rRNA data were performed using the R package Tax4Fun [23]. A closed reference feature table generated from QIIME2, utilizing the SILVA database, was applied for the analysis.

**Statistical analysis and visualization**

Statistical methods were depicted in figure legends. Pairwise comparisons were performed to evaluate differences in MicroAgeGap across different index, including dental caries status, periodontitis status, BMI classification, using the ‘pairwise.t.test’ function. The Bonferroni method was applied to adjust *p*-values for multiple comparisons.

Statistical results were described and visualized with Origin Software (2021b) and R software (v 4.0.3) using the R package ggplot2 (v 3.4.4), R package pheatmap (v 1.0.12).

**Supplementary Figures**





Figure S1. Alterations of the saliva microbiota across age groups

(A) Number of samples each age category.

(B) Number of samples categorized by disease and exposure states.

(C and D) Shannon diversity across age groups, shown in all the database (C) and stratified by continues (D). Boxplots display the 25th and 75th percentiles, the median, and values within 1.5 times the interquartile range. *P*-values were calculated with pairwise Wilcoxon tests and adjusted with the BH method.

(E and F) Average relative abundance of the most abundant genera across age groups, shown in all the database (E) and stratified by continues (F).





Figure S2. Performance of the trained microbial aging model and external validation

(A) Performance of the trained microbial aging model in the test set (*n* = 1,360, left) and distributions of MicroAgeGap (right) according to continents with LightGBM model.

(B) Performance of the trained microbial aging model in the test set of Asia, Europe, North America, and Oceania.

(C) External validation using PRJNA1162741 and distribution of age of train test and PRJNA1162741 (left). Performance of the trained microbial aging model in external validation database (PRJNA1162741, right).

(D) Performance of the trained microbial aging model in the test set of Healthy and Unhealthy.

(E) Performance of the trained microbial aging model in the test set of different conditions, including head and neck cancer, schizophrenia, asthma and COVID-19.

Correlation coefficients shown in Scatter plots were Pearson correlation coefficients. The differences in distribution of age were analyzed with two-sample Kolmogorov-Smirnov test. RMSE, root mean squared error; MAE, mean absolute error.





Figure S3. Microbial aging performance based on the other selected machine-learning Model algorithm

Performance of the trained microbial aging model in the test set (*n* = 1,360, left) and distributions of MicroAgeGap (right) according to continents with LASSO (A), XGBoost (B), Elastic Net (C), simple neural network (D) and deep neural network (E) model. Correlation coefficients shown in Scatter plots (right) were Pearson correlation coefficients. Violin plots (left) with center line, box limits and whiskers representing the median, interquartile range and minima/maxima within each group, respectively. RMSE, root mean squared error; MAE, mean absolute error.





Figure S4. The fluctuation of MicroAgeGap was correlated with the severity of oral diseases

(A and B) Associations between MicroAgeGap and different subtypes of dental caries (A) and periodontitis (B). The panels included horizontal bar plots depicting MSE of MicroAgeGap grouped by different subtypes (left), scatter plots showing the association between predicated MicroAge and actual age colored by subtypes (middle) and vertical bar plots representing the distribution of MicroAgeGap from different subtypes of dental caries or periodontitis (right).

(C) Association between MicroAgeGap and clinical examination indicators of periodontitis, including probe depth (PDD, right), and attachment loss (CAL, left). Rampant caries (RC); Rampant caries associated with high-sugar diet (RC-HD); Rampant caries associated with Sjögren’s syndrome (RC-SS); Periodontitis (PD); Human immunodeficiency virus, HIV; MSE, mean squared error; PDD, probe depth; CAL, attachment loss. *p* < 0.05; ** *p* < 0.01; *** *p* < 0.001. Statistical significance was calculated by Pairwise t-test test. The Bonferroni method was utilized to adjust *p*-values.





Figure S5. MicroAge in younger, elder age and centenarian samples

1. Distribution of age of train test and PRJEB25916.

(B) Performance of the trained microbial aging model in the younger age group (19-33 years) (left) and Associations between MicroAgeGap and BMI (right).

(C) Performance of the trained microbial aging model in the elder age group (68-88 years) (left) and Associations between MicroAgeGap and BMI (middle), number of drugs taken per day(right).

(D) Performance of the trained microbial aging model in the centenarians group (99-107 years) (left) and Associations between MicroAgeGap and BMI (middle), number of drugs taken per day(right).

Low body weight: BMI < 18.5; Normal body weight: BMI ∈ [18.5, 24.9]; Overweight: BMI ∈ [24.9, 29.9]; Obesity: BMI ≥ 30; MSE, mean squared error; Body mass index, BMI; RMSE, root mean squared error; MAE, mean absolute error. **p* < 0.05; ** *p* < 0.01; *** *p* < 0.001. Statistical significance was calculated by Pairwise t-test test. The Bonferroni method was utilized to adjust *p*-values.





Figure S6. Top 30 important features contributing to MicroAge predicted model

(A) Top 30 important features were selected according to their gain values of LightGBM model colored by genus.

(B) Associations between MicroAge predicted by the top 30 important features and actual ages from all the samples (*n* = 7,235).

(C) Multiple linear regression model was used to analyze the associations between MicroAgeGap (predicted by all the features (blue) or the top 30 important features (red)) and types of diseases and environmental status from all the samples. The beta estimates, along with their 95% confidence intervals, for the association between MicroAgeGap and each outcome were displayed on the *x*-axis.

(D) Network analysis of the relevance of the top 30 important features and other features in the test set of Asia, Europe, North America, and Oceania.

MSE, mean squared error; Corona Virus Disease 2019, COVID-19; Oral squamous cell carcinoma, OSCC; Human immunodeficiency virus, HIV.





Figure S7. The function prediction of top 30 important features across the lifespan

The potential functions of the top 30 important features were predicted using Tax4Fun annotated with KEGG Ortholog terms (Level 1: A; Level 2: B; Level 3: C) grouped by age groups and continents. Kyoto Encyclopedia of Genes and Genomes: KEGG.

**References**

1. Chen, M. Y., J. W. Chen, L. W. Wu, K. C. Huang, J. Y. Chen, W. S. Wu, W. F. Chiang, et al. 2021. “Carcinogenesis of Male Oral Submucous Fibrosis Alters Salivary Microbiomes.” *Journal of Dental Research* **100**: 397-405. <https://doi.org/10.1177/0022034520968750>

2. Iwasawa, Kentaro, Wataru Suda, Tomoyuki Tsunoda, Manari Oikawa-Kawamoto, Shuichiro Umetsu, Lena Takayasu, Ayano Inui, et al. 2018. “Dysbiosis of the salivary microbiota in pediatric-onset primary sclerosing cholangitis and its potential as a biomarker.” *Scientific Reports* **8**: 5480. <https://doi.org/10.1038/s41598-018-23870-w>

3. Khasnobish, Anushka, Lena Takayasu, Ken-ichi Watanabe, Tien Thi Thuy Nguyen, Kensuke Arakawa, Osamu Hotta, Kensuke Joh, et al. 2021. “Dysbiosis in the Salivary Microbiome Associated with IgA Nephropathy-‍A‍ ‍Japanese Cohort Study.” *Microbes and Environments* **36**. <https://doi.org/10.1264/jsme2.ME21006>

4. Morishima, Seiji, Kaori Takeda, Setsue Greenan, Yoshinobu Maki. 2022. “Salivary microbiome in children with Down syndrome: a case-control study.” *BMC Oral Health* **22**: 438. <https://doi.org/10.1186/s12903-022-02480-z>

5. Morton, James T., Clarisse Marotz, Alex Washburne, Justin Silverman, Livia S. Zaramela, Anna Edlund, Karsten Zengler, Rob Knight. 2019. “Establishing microbial composition measurement standards with reference frames.” *Nature Communications* **10**: 2719. <https://doi.org/10.1038/s41467-019-10656-5>

6. Patnaik, Santosh K., Eduardo G. Cortes, Eric D. Kannisto, Achamaporn Punnanitinont, Samjot S. Dhillon, Song Liu, Sai Yendamuri. 2021. “Lower airway bacterial microbiome may influence recurrence after resection of early-stage non-small cell lung cancer.” *The Journal of Thoracic and Cardiovascular Surgery* **161**: 419-429.e416. <https://doi.org/10.1016/j.jtcvs.2020.01.104>

7. Poole, Angela C., Julia K. Goodrich, Nicholas D. Youngblut, Guillermo G. Luque, Albane Ruaud, Jessica L. Sutter, Jillian L. Waters, et al. 2019. “Human Salivary Amylase Gene Copy Number Impacts Oral and Gut Microbiomes.” *Cell Host & Microbe* **25**: 553-564.e557. <https://doi.org/10.1016/j.chom.2019.03.001>

8. Shouval, Roni, Adi Eshel, Bar Dubovski, Amir A. Kuperman, Ivetta Danylesko, Joshua A. Fein, Shalev Fried, et al. 2020. “Patterns of salivary microbiota injury and oral mucositis in recipients of allogeneic hematopoietic stem cell transplantation.” *Blood Advances* **4**: 2912-2917. <https://doi.org/10.1182/bloodadvances.2020001827>

9. Stahringer, Simone S., Jose C. Clemente, Robin P. Corley, John Hewitt, Dan Knights, William A. Walters, Rob Knight, Kenneth S. Krauter. 2012. “Nurture trumps nature in a longitudinal survey of salivary bacterial communities in twins from early adolescence to early adulthood.” *Genome Research* **22**: 2146-2152. <https://doi.org/10.1101/gr.140608.112>

10. Yama, K., S. Morishima, K. Tsutsumi, R. Jo, Y. Aita, T. Inokuchi, T. Okuda, et al. 2024. “Oral Microbiota Development in the First 60 Months: A Longitudinal Study.” *Journal of Dental Research* **103**: 1249-1257. <https://doi.org/10.1177/00220345241272011>

11. Xu, Tiansong, Lihuang Yan, Bohui Sun, Qi Xu, Jieni Zhang, Wenhui Zhu, Qian Zhang, Ning Chen, Guoli Liu, Feng Chen. 2022. “Impacts of Delivery Mode and Maternal Factors on Neonatal Oral Microbiota.” *Frontiers in Microbiology* **13**: 915423. <https://doi.org/10.3389/fmicb.2022.915423>

12. Bolyen, Evan, Jai Ram Rideout, Matthew R. Dillon, Christian C. Abnet, Gabriel A. Al-Ghalith, Harriet Alexander, Eric J. Alm, et al. 2019. “Author Correction: Reproducible, interactive, scalable and extensible microbiome data science using QIIME 2.” *Nature Biotechnology* **37**: 1091-1091. <https://doi.org/10.1038/s41587-019-0252-6>

13. Quast, Christian, Elmar Pruesse, Pelin Yilmaz, Jan Gerken, Timmy Schweer, Pablo Yarza, Jörg Peplies, Frank Oliver Glöckner. 2013. “The SILVA ribosomal RNA gene database project: Improved data processing and web-based tools.” *Nucleic Acids Research* **41**: D590-D596. <https://doi.org/10.1093/nar/gks1219>

14. Ranstam, Jonas, Jonathan A. Cook. 2018. “LASSO regression.” *British Journal of Surgery* **105**: 1348-1348. <https://doi.org/10.1002/bjs.10895>

15. Chen, Tianqi, Tong He, Michael Benesty, Vadim Khotilovich, Yuan Tang, Hyunsu Cho, Kailong Chen, Rory Mitchell, Ignacio Cano, Tianyi Zhou. 2015. “Xgboost: extreme gradient boosting.” *R package version 0.4-2* **1**: 1-4.

16. Ke, Guolin, Qi Meng, Thomas Finley, Taifeng Wang, Wei Chen, Weidong Ma, Qiwei Ye, Tie-Yan Liu. 2017. “Lightgbm: A highly efficient gradient boosting decision tree.” *Advances in Neural Information Processing Systems* **30**.

17. Friedman, Jerome, Trevor Hastie, Rob Tibshirani, Balasubramanian Narasimhan, Kenneth Tay, Noah Simon, Junyang Qian, James Yang. 2023. “glmnet: Lasso and elastic-net regularized generalized linear models.” *Astrophysics Source Code Library*: ascl: 2308.2011.

18. Ripley, Brian, William Venables. 2016. “nnet: Feed-forward neural networks and multinomial log-linear models.” *R package version* **7**.

19. Gulli, Antonio, Sujit Pal. 2017. Deep learning with Keras. *Packt Publishing Ltd*,

20. Wang, Weijie, Yanmin Lu. 2018. Analysis of the mean absolute error (MAE) and the root mean square error (RMSE) in assessing rounding model. *IOP Conference Series: Materials Science and Engineering*;324:012049.

21. Hodson, Timothy O. 2022. “Root mean square error (RMSE) or mean absolute error (MAE): When to use them or not.” *Geoscientific Model Development Discussions* **2022**: 1-10.

22. Bastian, Mathieu, Sebastien Heymann, Mathieu Jacomy. 2009. Gephi: An Open Source Software for Exploring and Manipulating Networks. *Proceedings of the Third International Conference on Weblogs and Social Media, ICWSM 2009, San Jose, California, USA, May 17-20, 2009*.

23. Aßhauer, Kathrin P., Bernd Wemheuer, Rolf Daniel, Peter Meinicke. 2015. “Tax4Fun: Predicting functional profiles from metagenomic 16S rRNA data.” *Bioinformatics* **31**: 2882-2884. <https://doi.org/10.1093/bioinformatics/btv287>
